# Impact of social distancing on mental health during the COVID-19 pandemic: An urgent discussion

**DOI:** 10.1177/0020764020927047

**Published:** 2020-05-21

**Authors:** Matias Carvalho Aguiar Melo, Douglas de Sousa Soares

**Affiliations:** 1 Health Sciences Center, Universidade de Fortaleza, Fortaleza, Brazil; 2 Hospital de Saúde Mental Professor Frota Pinto, Fortaleza, Brazil

Dear Editors,

The coronavirus disease 2019 (COVID-19) outbreak has spread across all continents, causing major economic losses, reduced physical interaction and significant psychological distress (Remuzzi & Remuzzi, 2020). In a large-scale Chinese survey, high prevalence of depression and anxiety was detected during the pandemic (Qiu et al., 2020). Torales et al. (2020) have discussed the impact of the pandemic in mental health and provided information about previous experiences of epidemics and social isolation. According to the World Health Organization (WHO), social distancing represents an important strategy to prevent the increase in cases and deaths by COVID-19, leading to subsequent overburdening of the health care systems (Jacobson et al., 2020). Despite some evidence about the psychological impact of quarantine in previous situations, little is known about the consequences of social distancing for the hundreds of millions of people under lockdown in the current scenario (Brooks et al., 2020; Venkatesh & Edirappuli, 2020).

A US study investigated more than 10 million Google searches and assessed the changes in mental health search queries after stay-at-home measures. Topics related to anxiety, negative thoughts, sleep disturbances, and suicidal ideation increased dramatically before stay-at-home orders with a levelling of the curve after implementation (Jacobson et al., 2020). A British online qualitative study with 27 participants assessed five focus groups during the early stages of the social distancing measures (5–12 days post lockdown). The isolation resulted in significant negative impacts on mental health and well-being within a short time of policy implementation, mainly in those with low-paid or precarious employment. Reduced social interaction, economic losses, and routine changes led to psychological and emotional impact, as demotivation, loss of meaning and decreased self-worth (Williams et al., 2020).

Another research with 683 US adolescents carried out 2 weeks post lockdown showed that engagement in social distancing was not significantly associated with their mental health. However, specific motivations for social distancing were related to different mental problems. Youth whose motivation was to prevent illness or avoid judgements reported greater anxiety symptoms. Those who engaged in social distancing because a friend told them had more depressive symptoms (Oosterhoff et al., 2020).

We believe that social isolation will probably increase fear, anxiety symptoms, loneliness, and depressed mood. Humans are social beings, independent of nationality or cultural background, and maintaining isolation for a long period might create significant psychological distress. The economic burden of the pandemic, with millions of jobs lost, increase in poverty and inequality might accentuate these feelings. This impact may be ever stronger in developing countries, for infected patients or those who live in nursing facilities, the homeless and people with mental disorders. Strategies like online social and psychological support, as well as calling friends and family members, may mitigate these feelings, but not end them.

Discussing the psychological impact of this social isolation is necessary, since this is an unprecedented situation in recent human History, with almost no record of its consequences. To date, studies on this important topic are scarce and present significant biases. Given the magnitude of the pandemic and the number of people under lockdown, it is urgent to conduct more researches to clarify the effect of social distancing in short- and long-term mental health.
